# Activation of a dormant replication origin is essential for *Haloferax mediterranei* lacking the primary origins

**DOI:** 10.1038/ncomms9321

**Published:** 2015-09-16

**Authors:** Haibo Yang, Zhenfang Wu, Jingfang Liu, Xiaoqing Liu, Lei Wang, Shuangfeng Cai, Hua Xiang

**Affiliations:** 1 State Key Laboratory of Microbial Resources, Institute of Microbiology, Chinese Academy of Sciences, No. 1 West Beichen Road, Chaoyang District, Beijing 100101, China; 2 University of Chinese Academy of Sciences, Beijing 100049, China

## Abstract

The use of multiple origins for chromosome replication has been demonstrated in archaea. Similar to the dormant origins in eukaryotes, some potential origins in archaea appear to be inactive during genome replication. We have comprehensively explored the origin utilization in *Haloferax mediterranei*. Here we report three active chromosomal origins by genome-wide replication profiling, and demonstrate that when these three origins are deleted, a dormant origin becomes activated. Notably, this dormant origin cannot be further deleted when the other origins are already absent and *vice versa*. Interestingly, a potential origin that appears to stay dormant in its native host *H. volcanii* lacking the main active origins becomes activated and competent for replication of the entire chromosome when integrated into the chromosome of origin-deleted *H. mediterranei*. These results indicate that origin-dependent replication is strictly required for *H. mediterranei* and that dormant replication origins in archaea can be activated if needed.

## Abstract (web summary)

Archaea use multiple origins for chromosome replication, but some potential origins appear to be inactive during the genome replication. Here, Yang *et al*. show that when active origins are deleted from *Haloferax mediterranei*, a dormant origin is activated and essential, suggesting an origin-dependent replication.

## Graphical abstract

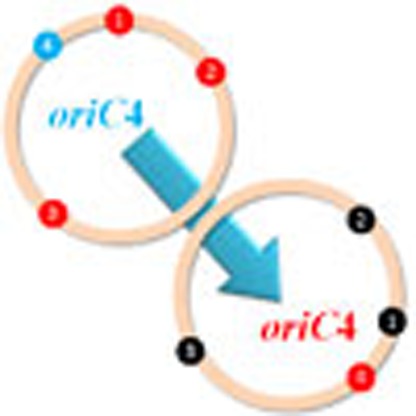

Chromosome replication starts at specific sites known as origins, where initiator proteins bind and recruit replication machinery components^1^,^2^. Bacteria use a single origin, which is usually adjacent to the initiator gene *dnaA*, to replicate their chromosome, whereas eukaryotes perform chromosome replication with multiple replication origins that are recognized by origin recognition complexes^2^,^3^. Dormant origins are replication origins that are not used in a normal cell cycle^4^. If DNA encounters replication stress or if adjacent origins fail to fire, dormant origins can be activated to help complete chromosome replication^4^,^5^,^6^, providing an important mechanism for cells to deal with replication stress and to maintain genomic stability^7^. For organisms with multiple replication origins on a chromosome, the coordination of origin utilization is vital to ensure complete and accurate genome duplication.

Chromosome replication using multiple replication origins has been widely recognized in archaea. The combination of an autonomously replicating sequence (ARS) assay or two-dimensional gel electrophoresis with marker frequency analysis (MFA) has led to the identification of multiple replication origins in *Sulfolobus* species^8^,^9^,^10^, haloarchaea^11^,^12^,^13^,^14^, *Aeropyrum pernix*^15^ and *Pyrobaculum calidifontis*^16^, thus greatly expanding our knowledge of archaeal replication origins. Archaeal replication origins generally contain an AT-rich region flanked by conserved repetitive DNA sequences designated as origin recognition boxes (ORBs)^17^. The initiator Cdc6 shows homology to both Orc1 and Cdc6 in eukaryotes and is responsible for ORB recognition and minichromosome maintenance (MCM) helicase recruitment^18^,^19^. As in bacteria, an archaeal replication origin usually lies upstream of the initiator gene *orc1/cdc6* (ref. 20). Studies in *Sulfolobus islandicus* and *Haloarcula hispanica* have demonstrated that the firing of specific origins is dependent on their adjacent initiators^10^,^21^, which facilitate the direct regulation of each origin. Based on the origin features described above, multiple *cdc6*-associated replication origins in 15 fully sequenced haloarchaeal genomes have been predicted^12^. However, not all of these origins were shown to be active in the genome-wide analysis of replication profiles^14^,^22^, similar to the presence of dormant origins in eukaryotes. However, no dormant origin has been experimentally identified in archaea.

The origin usage in one cell cycle was investigated in *Sulfolobus acidocaldarius*, which possesses three origins on its chromosome. The three replication origins fire once per cell cycle, with *oriC1* and *oriC3* firing slightly earlier than *oriC2* (ref. 23). For the three origins in *S. islandicus* and the two origins on the chromosome of *H. hispanica*, gene knockout experiments demonstrated that no single origin was essential, but at least one *orc1/cdc6* gene (for *S. islandicus*) or one origin (for *H. hispanica*) was required for chromosome replication^10^,^21^. Intriguingly, all active replication origins on the chromosome in *H. volcanii* H26 can be knocked out, and the resultant strain grows even faster than the parent strain^22^. Owing to the requirement for RadA recombinase, which is essential for homologous recombination, homologous recombination was proposed to account for replication initiation in this origin deletion strain of *H. volcanii*^22^.

In this study, the utilization of multiple replication origins and the consequences of origin deletion were investigated in *Haloferax mediterranei*, which belongs to the same genus as *H. volcanii*. We identified the replication origins on the chromosome and examined origin utilization throughout the growth phase. Surprisingly, knocking out all active origins on the chromosome led to the activation of a dormant replication origin, *oriC4-cdc6H*. The *oriC4* is essential for chromosome replication in the absence of all other active origins, and the three active origins cannot be deleted simultaneously if *oriC4* has been knocked out. Interestingly, when *oriC4-cdc6H* was replaced with *oriP2-orc13*, a putative dormant origin from *H. volcanii, oriP2-orc13* could also efficiently initiate the replication of the entire chromosome of *H. mediterranei* in the absence of all other active origins. These results demonstrate that dormant origins can be activated in archaea if needed, and that chromosome replication in *H. mediterranei*, unlike that in *H. volcanii*, strictly requires origin-dependent initiation.

## Results

### Coordinated utilization of three chromosomal origins

**Figure 1:**
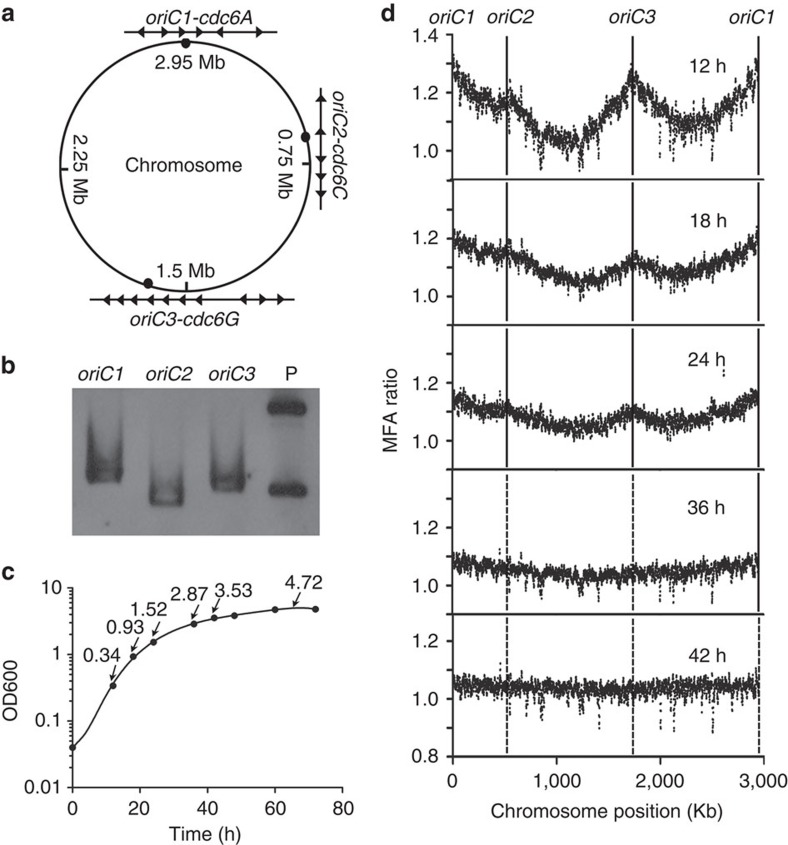
Three replication origins are utilized for chromosome replication in coordination with cell growth. (**a**) Prediction of the replication origins in the *H. mediterranei* chromosome. The three replication origins are indicated with filled ovals, and the corresponding ORB motifs are represented by triangles. (**b**) The ARS assay for the predicted origins. The corresponding fragments *oriC1-cdc6A*, *oriC2-cdc6C* and *oriC3-cdc6G* were cloned into the non-replicating plasmid pBI501 and the resulting plasmids pBI501*-oriC1-cdc6A*, pBI501*-oriC2-cdc6C* and pBI501-*oriC3-cdc6G* (Supplementary Table 1) were transferred into strains with the corresponding fragments deleted. Southern blot analysis was performed, using the *bla* gene as the probe. Lane P contains the plasmid pBI501**-***oriC3-cdc6G* isolated from *E. coli*, and the other lanes contain the crude DNA extracted from the corresponding *H. mediterranei* transformants. (**c**) The growth curve of *H. mediterranei* DF50. The time points at which the cultures were collected for MFA are indicated with arrows, and the corresponding OD600 values are labelled. (**d**) The utilization of replication origins throughout the growth phase. The chromosome replication profiles of *H. mediterranei* at 12, 18, 24, 36 and 42 h were generated by MFA. Ratios of the marker hybridization signals from the indicated time-point samples versus the stationary phase sample (66 h) are plotted against the chromosome position. The three replication origins *oriC1*, *oriC2* and *oriC3* are indicated at the top. The utilized origins are marked with solid lines, while the non-utilized origins are marked with dotted lines.

Genome sequencing has revealed that multiple initiator genes (*cdc6*) are encoded in the *H. mediterranei* genome, with eight in the main chromosome and five in the three megaplasmids^24^. Because replication origins are dependent on adjacent initiator proteins (Cdc6) for firing^10^,^21^, the replication origins of the *H. mediterranei* chromosome were predicted using Cdc6 and potential ORBs associated with these initiator genes^12^. In addition, as the genomes of *H. mediterranei* and *H. volcanii* share high homology over the entire chromosome, except for a 1-Mb fragment inversion between the two rRNA operons, the predicted origins and *cdc6* genes were also compared with those of *H. volcanii*. Of the eight *cdc6* genes in the *H. mediterranei* chromosome, *cdc6A*, *cdc6C* and *cdc6G* share high identity (>90%) with the three main replication initiator genes *orc1*, *orc2* and *orc5*, respectively, in *H. volcanii*. These three *cdc6* genes were also adjacent to inverted ORB repeats, representing a conserved characteristic of archaeal replication origins (Fig. 1a and Supplementary Fig. 1). Therefore, the three predicted *cdc6* genes with adjacent ORB-containing regions were designated as *oriC1-cdc6A*, *oriC2-cdc6C* and *oriC3-cdc6G* (Fig. 1a).

Genetic assays were performed to investigate the ARS activities of the three predicted origins. Three fragments, *oriC1-cdc6A*, *oriC2-cdc6C* and *oriC3-cdc6G*, were cloned into the non-replicating plasmid pBI501 (Supplementary Table 1), which cannot be replicated in any haloarchaea if no haloarchaeal ARS fragment is inserted into it. The resulting plasmids were transferred into *H. mediterranei* strains (Table 1) with the corresponding chromosome fragments deleted to avoid integration of the plasmids into the chromosome. As expected, the transformants were obtained. The presence of autonomously replicating plasmids in the corresponding transformants was determined by Southern blot analysis (Fig. 1b), which was further confirmed by retransferring the plasmids from *H. mediterranei* back into *Escherichia coli* (Supplementary Fig. 2). These results clearly showed that the three predicted origins were all capable of autonomous replication.

Whole-genome DNA microarray-based MFA was then used to examine the replication profile of *H. mediterranei* and the utilization of the three predicted origins throughout the entire growth phase. According to the growth curve, samples for DNA extraction were collected in the early exponential phase (12 h), middle exponential phase (18, 24 h), late exponential phase (36, 42 h) and stationary phase (66 h) (Fig. 1c). The MFA results for the 12-h sample revealed three peaks, suggesting that three origins were used to initiate chromosome replication. The three peaks, located proximal to *cdc6A*, *cdc6C* and *cdc6G*, represented *oriC1*, *oriC2* and *oriC3*, respectively (Fig. 1d). The variable heights of the three peaks could reflect different initiation efficiencies of these origins, indicating that *oriC1* is likely more efficient at all of the time points (Fig. 1d). The MFA peak heights of the origins decreased gradually from 12 to 24 h, but the pattern of the differential initiation efficiencies of the origins was maintained (Fig. 1d). At 36 h, only the peak corresponding to *oriC1* was slightly visible, probably because only a small percentage of cells were cycling at this time point. The profile of the 42-h sample showed no prominent peaks (Fig. 1d), which was consistent with strain growth near the stationary phase. Therefore, despite the different initiating efficiencies, the three replication origins were used in coordination with cell growth.

### Activation of a dormant origin

**Figure 2:**
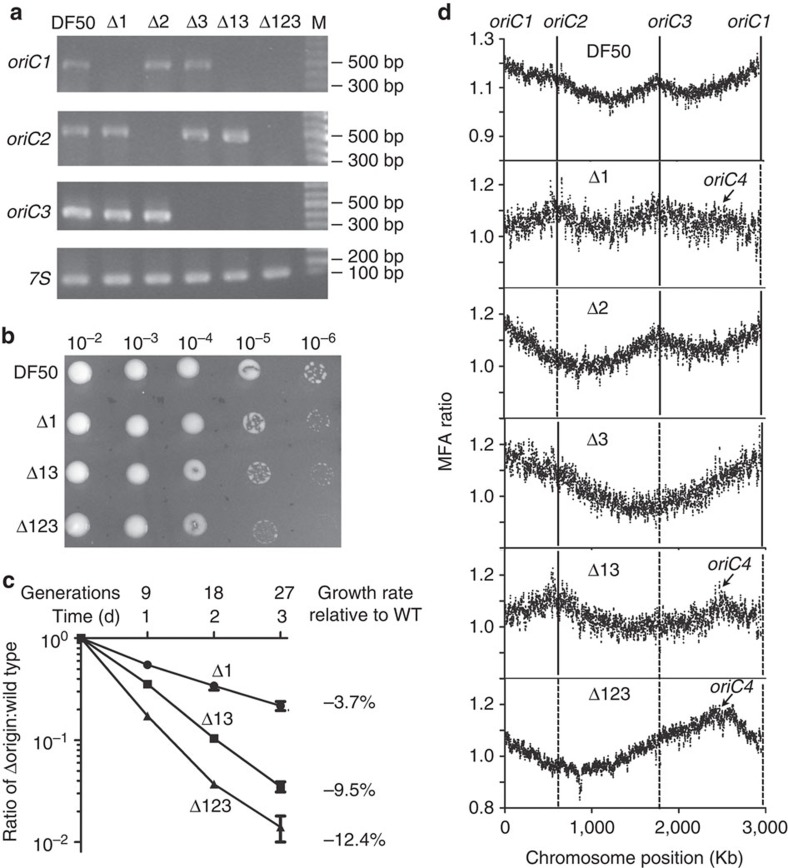
Activation of the dormant origin *oriC4* for chromosome replication in the origin deletion strains. (**a**) The origin deletion strains DF50Δ*oriC1* (Δ1), DF50Δ*oriC2* (Δ2), DF50Δ*oriC3* (Δ3), DF50Δ*oriC1*Δ*oriC3* (Δ13) and DF50Δ*oriC1*Δ*oriC2*Δ*oriC3* (Δ123) were confirmed by PCR analyses. The primers were specific for *oriC1*, *oriC2*, *oriC3* and *7S*; *7S* was used as an internal control. (**b**) The growth phenotypes of DF50Δ*oriC1*, DF50Δ*oriC1*Δ*oriC3* and DF50Δ*oriC1*Δ*oriC2*Δ*oriC3* compared with that of DF50. Serial dilutions of stationary phase cells (in equal amounts) were spotted onto AS-168 plates supplemented with uracil. (**c**) Pairwise growth competition assays comparing DF50 (wild-type) and origin deletants. The average with the s.e. of three parallel experiments is plotted. (**d**) Comparison of the chromosome replication profiles of DF50, DF50Δ*oriC1* (Δ1), DF50Δ*oriC2* (Δ2), DF50Δ*oriC3* (Δ3), DF50Δ*oriC1*Δ*oriC3* (Δ13) and DF50Δ*oriC1*Δ*oriC2*Δ*oriC3* (Δ123). The peaks corresponding to the activation of *oriC4* are indicated with arrows.

The above results demonstrated that three origins were used to replicate the chromosome, but with different initiating efficiencies. To investigate origin utilization when specific origins were deleted and to test the viability of the strain lacking all of these active origins on the chromosome, the three origins were targeted for knockout. Interestingly, knockout of each origin, double knockout of the *oriC1* and *oriC3* and simultaneous knockout of all three origins could be successfully obtained (Fig. 2a). These origin-deleted strains grew more slowly than the wild-type strain (Fig. 2b), with growth defect of ∼3.7% (for Δ*oriC1*) to 12.4% (for Δ*oriC1*Δ*oriC2*Δ*oriC3*) (Fig. 2c). However, the observed survival and growth of the DF50Δ*oriC1*Δ*oriC2*Δ*oriC3* strain indicated that efficient chromosome replication could still occur in the absence of the three main origins.

Microarray-based MFA was then used to examine the replication profile of strains with deletions of one, two or all three of the origins. In the single origin deletion strains, the peaks corresponding to the deleted origins disappeared (Fig. 2d), supporting the observation that the three origins on the chromosome were active. Notably, a new prominent peak of replication initiation emerged in the strain that had all three origins deleted, whereas the three peaks corresponding to the original origins disappeared. This new peak, located near the *cdc6H* gene, represented a new replication origin and was designated *oriC4* (Fig. 2d). Remarkably, the new peaks corresponding to *oriC4* were also observed in DF50Δ*oriC1*Δ*oriC3* and perhaps in DF50Δ*oriC1*, but not in DF50Δ*oriC2* or DF50Δ*oriC3* (Fig. 2d). Therefore, when the active origins on the chromosome were knocked out, a dormant replication origin was activated to replicate the entire chromosome either alone or in combination with other active origins. Interestingly, we noticed that the degree of activation or the initiation efficiency of *oriC4* was variable, as the peaks corresponding to *oriC4* in the replication profiles of the origin deletion strains had different heights (Fig. 2d). In the replication profile of DF50Δ*oriC1*, the *oriC4* peak was only slightly visible, whereas in that of DF50Δ*oriC1*Δ*oriC3* the *oriC4* peak was nearly the same height as the *oriC2* peak. Finally, in DF50Δ*oriC1*Δ*oriC2*Δ*oriC3*, the *oriC4* peak was the only peak and had a prominent height. Therefore, the activation of *oriC4* was positively correlated with the number of replication origins knocked out. To the best of our knowledge, this is the first report of the activation of a dormant origin in the domain of Archaea.

### The dormant *oriC4* depends on adjacent *cdc6H* for firing

**Figure 3:**
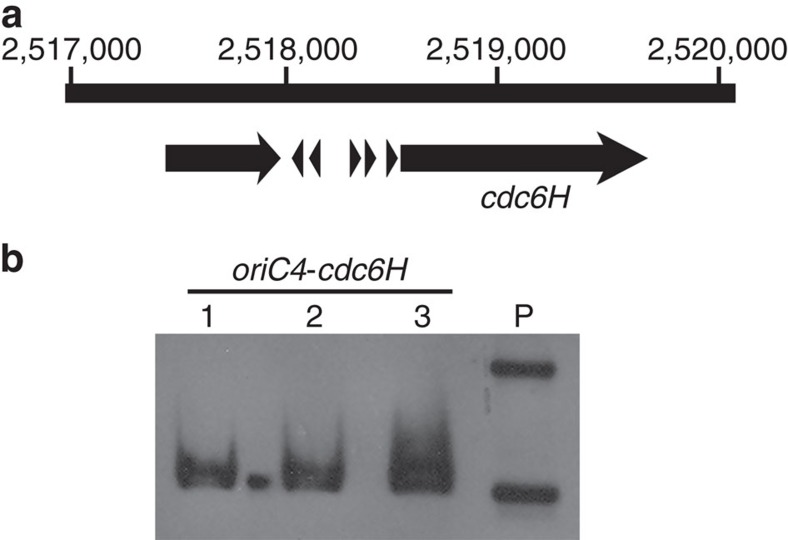
*oriC4* is dependent on *cdc6H* for firing. (**a**) Sequence features of the predicted location of *oriC4*. ORB motifs adjacent to the *cdc6H* gene are indicated with triangles. The numbering refers to the chromosomal position. (**b**) The ARS assay for *oriC4-cdc6H*. The fragments *oriC4*, *cdc6H* and *oriC4-cdc6H* were examined for autonomous replicating ability, using DF50Δ*oriC4-cdc6H* as the transformation host. Transformants were only obtained from the transformation corresponding to *oriC4-cdc6H*. Southern blot analysis was performed, using the *bla* gene as the probe. Lane P contains the plasmid pBI501**-***oriC4-cdc6H* (Supplementary Table 1) isolated from *E. coli*, whereas the other lanes (lane 1–3) contain crude DNA extracted from the *H. mediterranei* transformants.

The new peak, corresponding to *oriC4*, observed in the replication profile of DF50Δ*oriC1*Δ*oriC2*Δ*oriC3* was proximal to *cdc6H*; however, its precise location and the crucial elements responsible for its replication initiation remained undetermined. Interestingly, *cdc6H* was adjacent to inverted ORB repeats (within the predicted *oriC4*), as was observed for *cdc6A*, *cdc6C* and *cdc6G*, among the eight *cdc6* genes in the *H. mediterranei* chromosome (Fig. 3a and Supplementary Fig. 1). Therefore, *oriC4* was proposed to be dependent on *cdc6H* for firing, and an ARS assay was used to test this proposal. The fragments *oriC4*, *cdc6H* and *oriC4-cdc6H* were individually cloned into pBI501, and their autonomous replication abilities were examined via transformation of the DF50Δ*oriC4-cdc6H* strain. Although no transformants were observed for the single *oriC4* or *cdc6H* fragment, transformants corresponding to the *oriC4-cdc6H* fragment were obtained, and Southern blot analysis confirmed the presence of autonomous replicating plasmids in the transformants (Fig. 3b). Therefore, *oriC4-cdc6H* is a novel replication origin. Although *oriC4-cdc6H* is dormant in the chromosome of wild-type *H. mediterranei*, it is capable of autonomous plasmid replication and can be activated for chromosome replication in *H. mediterranei* in the absence of other active origins.

### Recent acquisition of *oriC4* may account for its dormancy

**Figure 4:**
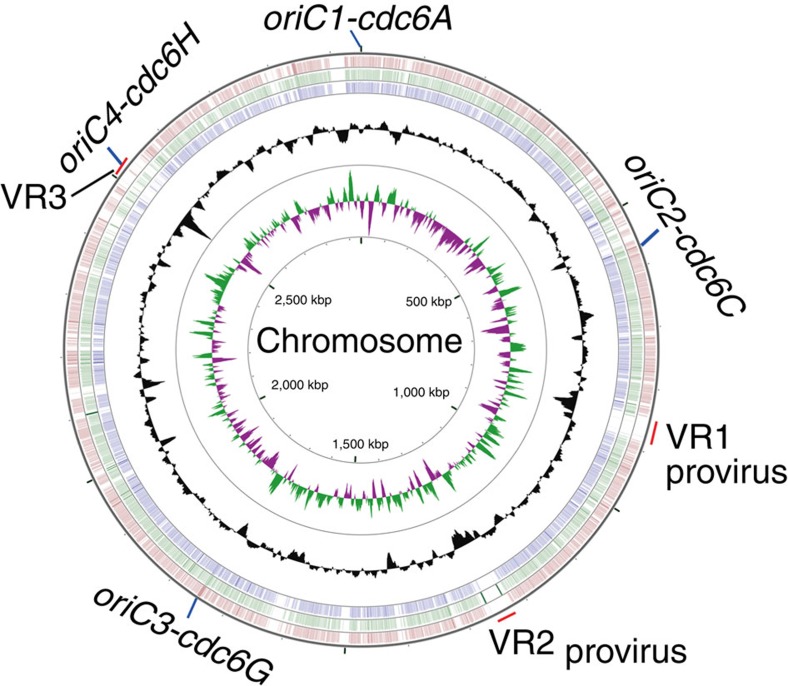
Comparative genomics of the replication origins of *Haloferax* species. Starting from the outside, the first, second and third circles represent the DNA sequence BLASTN hits of the *H. mediterranei* chromosome against *H. volcanii*, *Haloferax elongans* ATCC BAA-1513 and *H. alexandrinus* JCM 10717, respectively. The fourth and fifth circles represent the G+C content and GC skew of the *H. mediterranei* chromosome, respectively. The four replication origins and three large variable G+C content regions (VR1, VR2 and VR3) on the *H. mediterranei* chromosome are indicated. The origins *oriC1-cdc6A*, *oriC2-cdc6C* and *oriC3-cdc6G* are located in the conserved chromosomal region, whereas *oriC4-cdc6H* is located in a variable chromosomal region (VR3).

To determine the evolutionary difference between the dormant origin and the active origins, we analysed the distribution of *H. mediterranei*-type origins in the *Haloferax* genus by comparing Cdc6 from *H. mediterranei* against the other 14 species via BLAST. The three active origins *oriC1-cdc6A*, *oriC2-cdc6C* and *oriC3-cdc6G* were found to be conserved in these *Haloferax* species (Table 2). However, the dormant origin *oriC4-cdc6H* was much less conserved and was present only in *H. mediterranei* and two other species, namely *H. larsenii* and *H. elongans* (Table 2). Interestingly, sequence context analysis revealed that *oriC1-cdc6A*, *oriC2-cdc6C* and *oriC3-cdc6G* were located in the conserved chromosomal context, whereas *oriC4-cdc6H* was located in the variable chromosomal context (Fig. 4). The conservation of these three active replication origins indicated that these origins were present in the initial *Haloferax* in the evolutionary process. However, in contrast to these original active replication origins, the dormant origin *oriC4-cdc6H* was likely acquired more recently. We propose that the recently acquired *oriC4-cdc6H* may be less competitive in the recruitment of inherent replication initiation factors (such as MCM), which may contribute to its dormant state in the wild-type strain.

### The *oriC4* is essential in absence of other active origins

The dormant origin *oriC4* was activated for chromosome replication in the absence of the other three active origins. To verify the necessity and importance of *oriC4* in strains lacking the primary active origins, we attempted to knock out *oriC4* in the DF50Δ*oriC1*Δ*oriC2*Δ*oriC3* strain and in the DF50 and DF50Δ*oriC1*Δ*oriC3* strains. Although knocking out *oriC4* in DF50 and DF50Δ*oriC1*Δ*oriC3* was easy, we failed to knock out *oriC4* in DF50Δ*oriC1*Δ*oriC2*Δ*oriC3* after screening more than 600 FOA^r^ (5-fluoroorotic acid resistant) colonies from two independent experiments (Table 3). This result indicated that *oriC4* is essential for chromosome replication in the absence of the other three origins. The necessity of *oriC4* was also tested using gene disruption experiments. Because the archaeal replication origin depends on an adjacent initiator protein for firing, *oriC4* inactivation was performed by disrupting the *cdc6H* gene. Notably, the disruption of *cdc6H* in DF50 and DF50Δ*oriC1*Δ*oriC3* resulted in a large number of large colonies, whereas the disruption of *cdc6H* in DF50Δ*oriC1*Δ*oriC2*Δ*oriC3* only resulted in sporadic tiny transformants (Supplementary Fig. 3a). PCR analysis of the tiny transformants revealed both disrupted and intact *cdc6H* genes (Supplementary Fig. 3b). As *H. mediterranei* has multiple copies of its chromosome^25^, the heterozygosity of *cdc6H* genes in this disruption approach further supports the requirement for *oriC4* in the DF50Δ*oriC1*Δ*oriC2*Δ*oriC3* strain.

**Figure 5:**
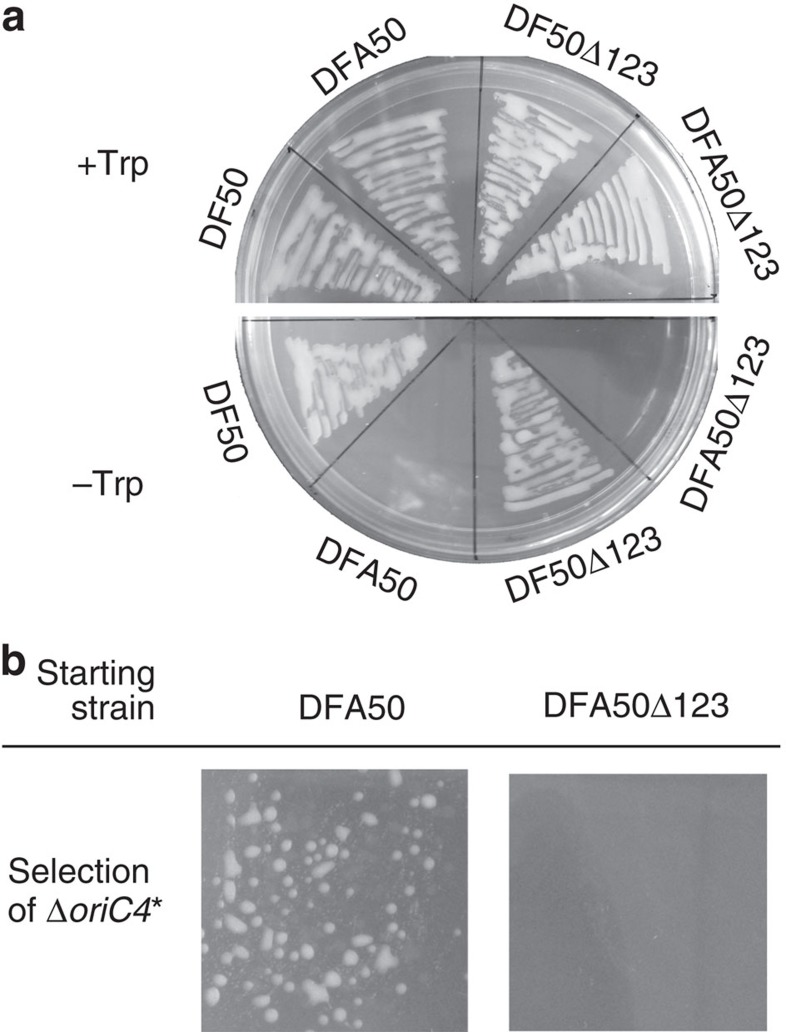
*oriC4* is essential for chromosome replication in the absence of the other three origins. (**a**) Cells of the DF50 and DF50Δ*oriC1*Δ*oriC2*Δ*oriC3* (DF50Δ123) strains and of the corresponding *trpA* knockout mutant strains, named DFA50 and DFA50Δ*oriC1*Δ*oriC2*Δ*oriC3* (DFA50Δ123), respectively, were streaked onto AS-168SY plates supplemented with uracil and with or without tryptophan. (**b**) For the positive selection of *trpA*-marked knockouts of *oriC4* in DFA50 and DFA50Δ*oriC1*Δ*oriC2*Δ*oriC3*, the corresponding propagated culture of one pop-in colony in the AS-168 broth was plated on AS-168SY plates supplemented with uracil and 5-FOA and without tryptophan. *The cell density of the corresponding culture of DFA50Δ*oriC1*Δ*oriC2*Δ*oriC3* for plating was 10 times that of DFA50.

To confirm the requirement for *oriC4* in DF50Δ*oriC1*Δ*oriC2*Δ*oriC3*, we also developed a *trpA*-marked positive selection system in *H. mediterranei* based on the *pyrF*-based gene knockout system^26^. First, *trpA* was knocked out to construct the tryptophan auxotrophic strains DFA50 and DFA50Δ*oriC1*Δ*oriC2*Δ*oriC3* (Fig. 5a). Then, *oriC4* was replaced with the *trpA* marker. Although we successfully established the *trpA*-marked knockout of *oriC4* in DFA50, we did not obtain any isolates for the *oriC4* knockout in DFA50Δ*oriC1*Δ*oriC2*Δ*oriC3* (Fig. 5b), reconfirming the requirement for *oriC4* in the absence of the other three origins. Remarkably, knocking out *oriC3* in DF50Δ*oriC1*Δ*oriC2*Δ*oriC4* and knocking out *oriC2* in DF50Δ*oriC1*Δ*oriC3*Δ*oriC4* were also unsuccessful. All of these results clearly indicate that origin-dependent initiation is strictly required for chromosome replication in *H. mediterranei* and that *oriC4* is essential for chromosome replication in the *H. mediterranei* DF50Δ*oriC1*Δ*oriC2*Δ*oriC3* strain.

Interestingly, *radA* expression was significantly higher in DF50Δ*oriC1*Δ*oriC2*Δ*oriC3* than in DF50 (Supplementary Fig. 4a). To test whether *radA* is essential in DF50Δ*oriC1*Δ*oriC2*Δ*oriC3*, as was observed in *H. volcanii* lacking all active origins^22^, *radA* was placed under the control of the inducible promoter p.tna, as described by Hawkins *et al*.^22^ Although the growth of DF50Δ*oriC1*Δ*oriC2*Δ*oriC3* was slower in the absence of RadA (when *radA* expression was not induced), unlike *H. volcanii*, RadA was not strictly required for its viability (Supplementary Fig. 4b). This finding further confirms that the chromosome replication of this origin-less *H. mediterranei* is origin dependent, which differs from the recombination-dependent replication recently proposed for *H. volcanii*^22^.

### Identification of the dormant origin *oriP1-cdc6L* on pHM500

**Figure 6:**
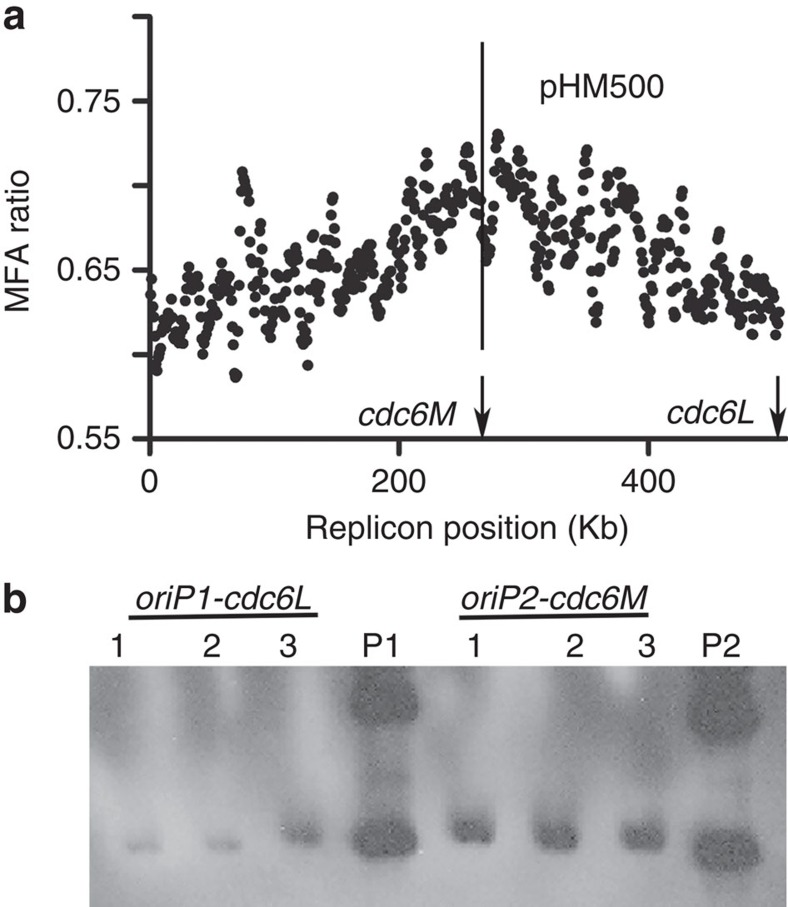
The prediction and identification of a dormant origin on pHM500. (**a**) The replication profile of pHM500. The active replication origin is indicated by a vertical line, and the positions of *cdc6L* and *cdc6M* are indicated by vertical arrows. (**b**) ARS assays for *oriP1-cdc6L* and *oriP2-cdc6M*. The plasmids pBI501-*oriP1-cdc6L* and pBI501**-***oriP2-cdc6M* (Supplementary Table 1) were transferred into strains with the corresponding fragments deleted. Southern blot analysis was performed, using the *bla* gene as the probe. Lanes P1 and P2 contain plasmids isolated from *E. coli* with pBI501**-***oriP1-cdc6L* for P1 and pBI501**-***oriP2-cdc6M* for P2, whereas the other lanes contain crude DNA extracted from the corresponding *H. mediterranei* transformants.

We also assessed extrachromosomal replicons in *H. mediterranei* to determine whether other dormant origins exist. Two *cdc6* genes encoded on pHM500, *cdc6L* and *cdc6M*, are adjacent to inverted ORB repeats, indicating that both genes are associated with replication origins (Supplementary Fig. 1). However, the pHM500 replication profile in the wild-type strain displayed only one peak located adjacent to *cdc6M* (Fig. 6a), indicating that *oriP2-cdc6M* is the active origin for pHM500 replication in the wild-type strain. We hypothesized that *oriP1-cdc6L*, acting as a dormant origin in the wild-type strain, would be activated for replication in the absence of *oriP2-cdc6M*. To test this hypothesis, we generated mutants lacking *oriP1-cdc6L* or *oriP2-cdc6M*; the autonomous replication abilities of the two origins were confirmed using an ARS assay (Fig. 6b and Supplementary Fig. 5a), indicating that either origin could be used to replicate the replicon in the absence of the other origin. In addition, our attempt to knock out *oriP1-cdc6L* in Δ*oriP2-cdc6M* was unsuccessful, and the disruption of *cdc6L* in Δ*oriP2-cdc6M* resulted in only a few tiny colonies (Supplementary Fig. 5b, 5c), indicating that *oriP1-cdc6L* is essential in the absence of *oriP2-cdc6M*. Therefore, we proposed that the dormant origin *oriP1-cdc6L* would be activated for pHM500 replication in the absence of *oriP2-cdc6M*.

### Heterogeneous *oriP2-orc13* initiates chromosome replication

**Figure 7:**
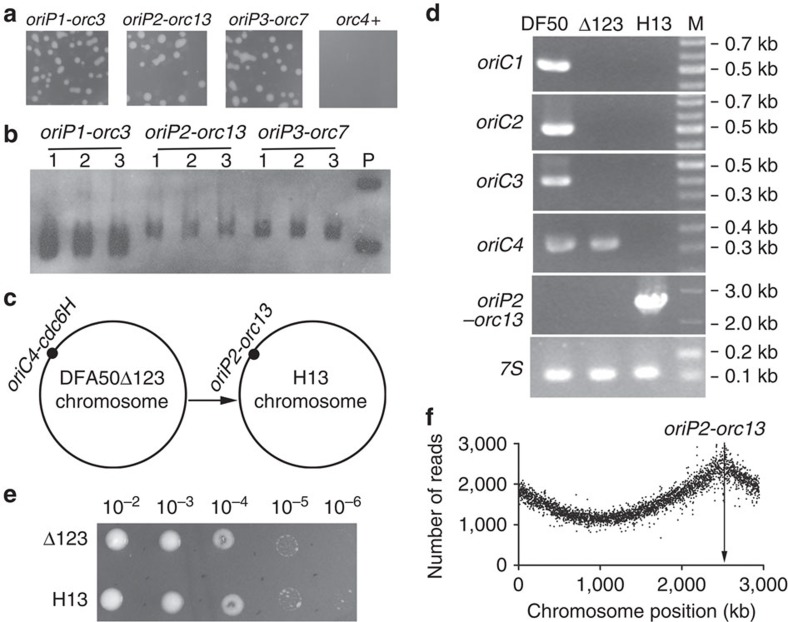
The dormant origin *oriP2-orc13* from *H. volcanii* can initiate the replication of the entire *H. mediterranei* chromosome. (**a**) ARS assays for *H. volcanii oriP1-orc3*, *orc4+* and the potential dormant origins *oriP2-orc13* and *oriP3-orc7*. Plasmids pBI501**-***oriP1-orc3*, pBI501**-***oriP2-orc13*, pBI501**-***oriP3-orc7*and pBI501**-***orc4+* (Supplementary Table 1) were transferred into DF50Δ*oriP2-cdc6M.* Fragment *orc4+* refers to the *orc4* gene plus the adjacent upstream intergenic region. Colonies were observed after 3 days at 37 °C. (**b**) Southern blot analysis using the *bla* gene as the probe. Lane P contains the plasmid pBI501**-***oriP3**-**orc7* isolated from *E. coli*, whereas the other lanes contain crude DNA extracted from the corresponding *H. mediterranei* transformants. (**c**) Schematic diagram of the replication origin of the *H. mediterranei* H13 chromosome. H13 was constructed by replacing the *oriC4-cdc6H* region in DFA50Δ*oriC1*Δ*oriC2*Δ*oriC3* (DFA50Δ123) with the *oriP2-orc13* region from *H. volcanii*. (**d**) The origin-replaced mutant H13 was confirmed by PCR analyses, with DF50 and DF50Δ*oriC1*Δ*oriC2*Δ*oriC3* (Δ123) as controls. The primers were specific for *oriC1*, *oriC2*, *oriC3*, *oriC4*, *oriP2-orc13* and *7S*; *7S* was used as an internal control. (**e**) The growth phenotype of H13 compared with DF50Δ*oriC1*Δ*oriC2*Δ*oriC3* (Δ123). Serial dilutions of stationary phase cells (equal amounts) were spotted onto AS-168 plates supplemented with uracil. (**f**) Marker frequency distribution for the H13 chromosome. High-throughput sequencing was performed for the total DNA from the exponential phase of H13, and the number of reads was calculated for every 1-kb window and plotted against the chromosome position. The position of the replication origin *oriP2-orc13* is marked with an arrow.

The replication origins of *H. volcanii* have been extensively characterized^11^,^22^, but no dormant origins have been reported in previous investigations. In the case of *H. mediterranei*, the two identified dormant origins *oriC4-cdc6H* and *oriP1-cdc6L* displayed the common features that both initiator genes were adjacent to the inverted ORB motifs and that no peaks of replication initiation emerged at the corresponding locations in the replication profiles of the wild-type strain. When these features were used to evaluate the potential origins in the *H. volcanii* main chromosome and in pHV4, which is integrated into the main chromosome of *H. volcanii* H26 (ref. 22), three *orc1/cdc6* genes associated with adjacent intergenic regions were found to fulfil these criteria: *orc4* on the main chromosome, and *orc13* and *orc7* on pHV4 (refs 12, 22) (Supplementary Table 2). Although no initiating activity was detected for these three fragments (putative origins) in the replication profiles of the origin-deleted *H. volcanii*^22^, we wondered whether they could be activated to replicate the *H. mediterranei* chromosome, given that dormant origins can be activated in *H. mediterranei* if needed. First, we tested the autonomous replicating abilities of the three fragments with an ARS assay in *H. mediterranei* and used the active origin *oriP1*-*orc3* as a positive control. In this assay, the DF50Δ*oriP2-cdc6M* strain was selected as the transformation host because *orc3* and *cdc6M* share high identities (98%), whereas no *cdc6* gene in *H. mediterranei* shares high identity with *orc4*, *orc13* or *orc7* (<55%). Consistent with the results obtained for *H. volcanii*^11^, no transformants were observed for the corresponding *orc4* fragment transformation. However, the other three fragments, *oriP1*-*orc3*, *oriP2*-*orc13* and *oriP3*-*orc7*, had autonomous replicating ability in *H. mediterranei* (Fig. 7a,b).

To test the initiating activity of the putative dormant origins from *H. volcanii* for chromosome replication in *H. mediterranei*, we attempted to replace the only origin (*oriC4-cdc6H*) on the chromosome of *H. mediterranei* DFA50Δ*oriC1*Δ*oriC2*Δ*oriC3* with the *H. volcanii oriP2-orc13* origin. Considering the autonomous replicating ability of *oriP2-orc13*, we expected that knocking in the *oriP2-orc13* fragment into the chromosome via a plasmid vector would be difficult. Thus, a linear DNA fragment containing *oriP2-orc13* and a *trpA* marker (for positive selection of the replacement) was used for gene replacement. Replacement of the *oriP2-orc13* origin at the *oriC4-cdc6H* position (Fig. 7c) was successfully conducted, as confirmed by both PCR (Fig. 7d) and Southern blot analysis (Supplementary Fig. 6), and the mutant strain was designated *H. mediterranei* H13 (Fig. 7c). Growth of the H13 strain containing only the *oriP2-orc13* replication origin was comparable to that of DF50Δ*oriC1*Δ*oriC2*Δ*oriC3* (Fig. 7e). Notably, the genome-wide replication profile of H13 revealed that chromosome replication was initiated only from *oriP2-orc13* (Fig. 7f). Thus, the putative dormant origin *oriP2-orc13* from *H. volcanii* can be used to efficiently replicate the entire chromosome of *H. mediterranei* in the absence of the other active origins.

## Discussion

The initiation of chromosome replication from multiple origins is very common in archaea. Based on the conserved characteristics of archaeal origins, multiple *cdc6*-associated replication origins have been predicted in 15 fully sequenced haloarchaeal genomes^12^. However, not all of these predicted origins are active, as revealed by genome-wide MFA^14^,^22^. Therefore, it is likely that dormant replication origins are widely distributed in haloarchaea. Because dormant origins can be activated for replicon replication in the absence of other active origins, they can act as backup sites for replication initiation when surrounding origins are inactivated or when replication forks stall, as has been demonstrated for dormant origins in eukaryotes^27^,^28^. In contrast to active origins, dormant origins in haloarchaea were proposed to have been recently acquired (Fig. 4), possibly from environmental plasmids, viruses or other haloarchaea. Interestingly, similar to our results that the recently acquired dormant origin is competent to replicate the entire chromosome of the origin-deleted *H. mediterranei*, suppression of the initiation defect of chromosome replication (for example, in the *dnaA* or *oriC*-deleted mutants) by plasmid or prophage integration has also been reported in bacteria^29^,^30^. As horizontal gene transfer among haloarchaea is common and the acquisition of replication origins is favourable for the transfer of new genetic content^31^,^32^, we propose that the acquired origins that accompanied foreign genetic contents are important for haloarchaea to shape the chromosomal structure and to adapt to harsh and variable environments. In addition, these recently acquired origins can be active or dormant as a result of different intracellular and extracellular conditions; this may act as an additional adaptive feature for these haloarchaea.

Interestingly, *oriC4* is dormant in wild-type *H. mediterranei* but is significantly activated in DF50Δ*oriC1*Δ*oriC2*Δ*oriC3*. In eukaryotes, competition among origins for limiting replication factors such as CDC45, Sld2 and Sld3 determines their initiation time and efficiency^33^,^34^. Because the *H. mediterranei* chromosome is polyploid^25^, as observed in other haloarchaea^35^, and all origins share the common helicase MCM, MCM may be one of the limiting factors, as previously proposed for *H. volcanii*^22^. Therefore, we can speculate that the ability of *oriC4-cdc6H* to recruit MCM may be weaker than that of other active origins, resulting in the dormant state of *oriC4* in the wild-type strain. The deletion of active origins would liberate more MCM molecules, allowing Cdc6H to recruit MCM and initiate replication at *oriC4*. The gradually increased activation of *oriC4-cdc6H* in the origin deletion strains supports this hypothesis (Fig. 2d). In particular, the firing ability of *oriC4* appears to be suppressed by *oriC1*, the most efficient origin on the chromosome, as *oriC4* could be slightly activated in the absence of *oriC1*. Interestingly, according to several phylogenetic analyses of Cdc6, Cdc6A is the most conserved Cdc6 and is distributed among all haloarchaeal genomes as well as other archaeal genomes^12^,^36^. Comparative genomic analysis has indicated that *oriC4-cdc6H* was recently acquired by *H. mediterranei* (Fig. 4). Therefore, the recent acquisition of *oriC4-cdc6H* may impair its ability to recruit MCM compared with the other active origins, which are products of a longer period of evolution.

Although the activity of each origin was different, the three active origins of *H. mediterranei* displayed a similar dynamic pattern in the chromosomal replication profiles throughout the growth phase (Fig. 1d). This finding indicates that mechanisms exist for regulating utilization of the multiple origins. For bacteria with a single origin on the chromosome, intiation regulation depends on the availability and activity of DnaA at the origin^2^. For archaea with multiple origins on the chromosome, the corresponding initiators may have similar mechanisms for the regulation of initiation. In *S. acidocaldarius*, transcription of the three initiator genes associated with the origins showed the strongest cell cycle-specific induction around the G1 phase^10^,^37^, which is important for initiator binding at the origins and for replication initiation. Gene expression analysis of *H. mediterranei* revealed that the transcription of all origin-associated *cdc6* genes on the chromosome was decreased in the stationary phase compared with the exponential phase^38^; this finding is consistent with the active utilization of origins during the exponential phase. Apart from the usage of distinct initiator proteins, archaeal replication origins (including the dormant origins) in one cell share common MCM and GINS^39^, and other unknown factors^40^. These factors may interact with the distinct *oriCs* and Cdc6s with different efficiencies, thereby coordinating the firing or inactivation of distinct origins, as discussed above. More importantly, multiple origins acting as a group may also be regulated by these factors via the same mechanisms to ensure the coordination of DNA replication with cell growth.

Notably, although both *H. mediterranei* and *H. volcanii* belong to the *Haloferax* genus, share high homology over the entire chromosome and grow well after the deletion of all known active origins, we demonstrated that the growth of *H. mediterranei* lacking known active origins is dependent on the activation of a dormant origin. It has been proposed that the *H. volcanii* H26 lacking known active origins may initiate replication using a recombination-dependent mechanism^22^. Importantly, our results demonstrated that activation of the dormant origin is essential for chromosome replication in the absence of the other active origins. This is consistent with results observed for other archaea such as *S. islandicus* and *H. hispanica*, in which at least one origin is required for chromosome replication^10^,^21^,^41^. For *H. volcanii* H26 (ref. 22), Michel and Bernander^41^ have proposed an alternative explanation, suggesting that dormant or newly integrated origins may be responsible for the viability of *H. volcanii* strains lacking the main replication origins; however, no experimental data are available yet to support this speculation. In this study, we have provided the first evidence that a dormant replication origin can be activated and become essential for chromosome replication on the deletion of all other primary origins. Interestingly, although *oriP2-orc13* and *oriP3-orc7* in *H. volcanii* were previously shown to stay dormant in the absence of other active origins^22^, these origins exhibited ARS activities in *H. mediterranei* (Fig. 7a,b), and at least *oriP2-orc13* (*oriP3-orc7* has not yet been tested) was able to initiate replication of the entire *H. mediterranei* chromosome without other active origins (Fig. 7f). It is possible that *H. mediterranei* but not *H. volcanii* may encode factors that are important for the activities of *oriP2-orc13* and *oriP3-orc7* or that *H. volcanii* may encode suppressive factors for these putative origins. Difference also exists for the essentiality of RadA for the origin-deleted *H*. *mediterranei* and *H. volcanii*. RadA is strictly required for *H. volcanii* in the absence of all active origins^22^. In contrast, although the growth of DF50Δ*oriC1*Δ*oriC2*Δ*oriC3* was slowed in the absence of RadA and *radA* gene expression in DF50Δ*oriC1*Δ*oriC2*Δ*oriC3* was significantly increased, RadA was not strictly required for the viability of *H*. *mediterranei* in which the main origins had been deleted (Supplementary Fig. 4). This further supports the notion that activation of the dormant origin but not recombination-dependent initiation is responsible for genome replication in *H. mediterranei* DF50Δ*oriC1*Δ*oriC2*Δ*oriC3*. Surprisingly, *H. volcanii* H26 lacking the known active origins exhibits accelerated growth^22^, whereas *H. mediterranei* lacking the main origins exhibits slightly slower growth (Fig. 2b,c and Fig. 7e). The reason for this growth discrepancy is unknown, but all of these results indicate that there might be substantial differences between *H. volcanii* and *H. mediterranei*, even though they belong to the same genus.

In summary, our characterization of origin utilization in *H. mediterranei* reveals for the first time that a dormant replication origin can be activated and becomes essential for chromosome replication in the absence of the other active origins in archaea. This further supports the notion that chromosome replication may generally require at least one replication origin. In addition, our results also suggest that replication initiation from multiple origins (including dormant ones) could be extensively regulated, thus providing a model system for investigating the regulatory mechanisms of Cdc6-dependent replication initiation. The observed difference between origin-less *H. mediterranei* and *H. volcanii* in the dependence on dormant origin activation may permit the identification of factors that are important for origin activity in haloarchaea.

## Methods

### Strains and plasmids

The *H. mediterranei* strains used in this study are listed in Table 1, and the constructed plasmids and corresponding oligonucleotides are listed in Supplementary Table 1. The plasmids used for gene knockout and gene disruption were derived from pHFX^26^, and the plasmids used for the ARS assay were derived from pBI501 (Supplementary Table 1). *E. coli* JM109 and JM110 cells were grown in Luria–Bertani medium containing 100 μg ml^−1^ ampicillin at 37 °C. The former strain was used for constructing plasmids, and the latter strain was used for preparing demethylated plasmids for the transformation of *H. mediterranei*^42^. *H. mediterranei* strains were cultivated at 37 °C in the nutrient-rich medium AS-168 (ref. 12) or in AS-168SY (AS-168 medium without yeast extract). When required, uracil, 5-FOA and tryptophan were added to the medium at final concentrations of 50 μg ml^−1^, 250 μg ml^−1^ and 50 μg ml^−1^, respectively.

### DNA preparation

The genomic DNA used for microarray analysis or high-throughput sequencing was prepared using the phenol–chloroform method^21^. The DNA was resuspended in Tris-EDTA buffer and stored at −20 °C. The crude genomic DNA used for Southern blot analysis was extracted by directly lysing *H. mediterranei* transformants with 200 μl double-distilled H_2_O and 100 μl phenol–chloroform, and the liquid supernatant was collected for the following analysis.

### Whole-genome DNA microarray-based MFA

Whole-genome DNA microarrays (12 × 135 K) were designed and manufactured by CapitalBio (Beijing, China) and Roche NimbleGen (Madison, USA), respectively, according to the whole genomic sequence of *H. mediterranei*^24^. Approximately 34 50-mer probes per kb, encompassing the entire genomic sequence, were designed.

Genomic DNA was extracted from the *H. mediterranei* cultures at specific time points, as indicated in Fig. 1c, or during the exponential phase (OD_600_≈0.5) and the stationary phase (OD_600_≈4.0), for the origin deletion strains. The exponential phase and stationary phase genomic DNA was then labelled with Cy3 and Cy5 (NimbleGen Dual-Colour Labelling Kit), respectively, according to the NimbleGen protocol. Array hybridization, washing and scanning and data normalization were performed as described in the study by Forsberg *et al*.^43^ The ratios were calculated as exponential phase values versus stationary phase values for each probe, and outliers (ratio >2 or <0.5) were excluded from the analyses. For each analysis, the excluded outliers accounted for <0.4% of the total probes; thus, ∼34 valid probes per kb remained. Owing to the high density of the probes, the ratios of the 30 probes were averaged as 1 point located at the first probe. The MFA graph was plotted with a 5-point window and a 1-point slide.

### High-throughput DNA sequencing and data analysis

Owing to the lack of probes specific for *oriP2-orc13*, the replication profile of *H. mediterranei* H13 was measured by high-throughput DNA sequencing. The genomic DNA was extracted from *H. mediterranei* H13 culture during the exponential phase (OD_600_≈0.35). Library preparation and sequencing with Illumina HiSeqTM 2500 were performed by Biomarker Technologies (Beijing, China), following Illumina protocols (San Diego, CA). Clean reads recovered after filtering low quality reads and artificially redundant reads were mapped to the *H. mediterranei* genome with the Burrows–Wheeler Alignment tool^44^. The number of reads was calculated for every 1-kb window and plotted against the chromosome position.

### *pyrF* marker-based genetic manipulation

The gene knockout experiments were conducted according to previously described procedures^26^. Briefly, two fragments (500–800 bp) located upstream and downstream of the target gene or fragment were amplified from *H. mediterranei* genomic DNA and ligated onto the pHFX plasmid. The constructed plasmids were transferred into the *H. mediterranei* strains. The polyethylene glycol-mediated transformation method^45^ was used, and the knockouts were achieved via double-crossover homologous recombination. When the knockout strains were screened, 5-FOA^r^ colonies (FOA^r^ indicates that the second homologous recombination has occurred) were selected on AS-168SY plates supplemented with uracil and 5-FOA. PCR analysis was then used to screen the knockout mutants.

Gene disruption was achieved via the single-crossover method. Briefly, a ∼500-bp fragment in the middle of the target gene was amplified and inserted into the pHFX plasmid. The constructed plasmids were transferred into *H. mediterranei* to disrupt the target gene through single-crossover homologous recombination.

Replacement of the *radA* promoter with the inducible promoter p.tna was conducted similar to the gene knockout, with minor modifications. Briefly, the upstream and downstream fragments of the *radA* promoter were placed flanking the p.tna fragment. The resultant plasmid pBI501-ptnaradA (Supplementary Table 1) was transferred into *H. mediterranei* strains to obtain the recombinant strain.

### *trpA* marker-based genetic manipulation

The *trpA*-marked positive selection system was constructed based on the *pyrF*-based gene knockout system^26^. First, the tryptophan auxotrophic strain DFA50 was constructed by knocking out the *trpA* gene with the plasmid pHFX-Δ*trpA* (Supplementary Table 1). Then, the *trpA* open reading frame under the *hsp5* promoter^46^ was inserted into pBI501 to obtain pHFA101.

For the knockout of *oriC4*, the upstream and downstream fragments of *oriC4* were placed flanking *P*_*hsp5*_*-trpA*. The resulting plasmid pHFA101Δ*oriC4* was transferred into DFA50, and a pop-in event was verified by PCR analysis. For positive selection of the pop-out event, the propagated culture of one pop-in colony in AS-168 broth was plated on AS-168SY plates supplemented with uracil and 5-FOA but without tryptophan. The *oriC4* knockouts could be selected on these plates because of the replacement of *oriC4* with *P*_*hsp5*_*-trpA*.

For the replacement of *oriC4-cdc6H* on the DFA50Δ*oriC1*Δ*oriC2*Δ*oriC3* chromosome with *oriP2-orc13*, the upstream and downstream fragments of *oriC4-cdc6H* were placed flanking a *trpA-oriP2-orc13* fragment (Supplementary Table 1). The Δ*oriC4-cdc6H*::*trpA-oriP2-orc13* cassette was then cut from the resultant plasmid pHFA101-Δ*oriC4-cdc6H*::*oriP2-orc13* and transferred into DFA50Δ*oriC1*Δ*oriC2*Δ*oriC3*. Transformants were selected on AS-168SY plates supplemented with uracil but without tryptophan to ensure the replacement of *oriC4-cdc6H* with *trpA-oriP2-orc13*.

### ARS assay

The ARS assay experiments were performed as previously described, with some modifications^12^. The target fragment was amplified and cloned into the non-replicating plasmid pBI501 (Supplementary Table 1), which could not be replicated in *H. mediterranei* unless an ARS was inserted. To avoid integration of the plasmids into the chromosome, the resulting plasmids were transferred into *H. mediterranei* strains (*pyrF*^-^) with the corresponding fragments deleted. If the cloned fragments could confer autonomous replication to the plasmid pBI501 with a *pyrF* selection marker gene, the transformants could be obtained on an AS-168SY plate without uracil. Plasmid recovery in the transformants, indicating the ARS ability of the cloned fragment, was verified using Southern blot analysis, as previously described^12^. This was further confirmed by retransferring the plasmids from *H. mediterranei* back to *E. coli.* As the plasmids for the ARS assay contained a replication origin of an *E. coli* plasmid, the corresponding *E. coli* transformants will and can only be obtained in this retransformation if the plasmids can autonomously replicate in *H. mediterranei* and can thus be isolated from the *H. mediterranei* transformants.

Full-length, uncropped Southern blot images are provided in Supplementary Fig. 7.

### Cell growth comparison and pairwise growth competition

To compare growth of the *H. mediterranei* DF50 (wild type) and the origin-mutant strains on solid plates, a serial dilution spotting assay was performed. Briefly, the wild-type and mutant strains were grown in AS-168 broth supplemented with uracil to the stationary phase (OD_600_≈4.0). The cell densities of the strains were adjusted to be equal before 5 10-fold serial dilutions were made with AS-168 broth (10^−2^ to 10^−6^). Then, 5 μl of each dilution was spotted on an AS-168 plate supplemented with uracil. Photographs were taken 2 to 3 days after spotting.

To compare growth in liquid culture, pairwise growth competition assays were performed as previously described^47^, with minor modifications. Briefly, the *H. mediterranei* DF50 and the origin-mutant strains (1:1 ratio, and ∼4 × 10^6^ for each) were mixed and inoculated in 10 ml AS-168 broth supplemented with uracil. The mixed culture was cultivated for ∼1 day to a density of ∼10^8^ cells per ml, and 5 × 10^6^ cells were then inoculated in 10 ml of the same fresh medium and cultured for an additional day; this process was repeated once. To count the cells, the culture from each time point was diluted, and 150 μl aliquots were plated on AS-168 plates supplemented with uracil. The wild-type and origin-mutant cells were identified by PCR, and the ratio was calculated based on >120 random colonies.

### Prediction and distribution of replication origins

The intergenic region immediately upstream of *cdc6* was assessed for the presence of consensus repeats with MEME software^48^ (motif size: 18-35; OMOPS model). The consensus repeats were manually evaluated, and those with a ‘G-string'^11^ were selected as the ORB elements. Only the intergenic regions with inverted ORB elements were considered as replication origins. To analyse the distribution of *H. mediterranei*-type replication origins in the *Haloferax* species, the BlastP programme (BLOSUM62 matrix) was used, comparing origin-associated Cdc6 proteins from *H. mediterranei* with those from certain *Haloferax* genomes (http://blast.ncbi.nlm.nih.gov/)^49^. Only those Cdc6 proteins with identities >80% (ref. 12) were recognized.

### Genome resources and comparative genomics

Two completed genomes, *H. mediterranei* ATCC 33500 and *H. volcanii* DS2 (ref. 50), and the contigs of 13 draft genomes, *H. sulfurifonti* ATCC BAA 897 (ref. 51), *H. mucosum* ATCC BAA 1512 (ref. 51), *H. denitrificans* ATCC 35960 (ref. 51), *H. prahovense* DSM 18310, *H. larsenii* JCM 13917, *H. gibbonsii* ATCC 33959, *H. elongans* ATCC BAA-1513, *H. alexandrinus* JCM 10717, *H. lucentense* DSM 14919, *Haloferax* sp. ATCC BAA-644, *Haloferax* sp. ATCC BAA-645, *Haloferax* sp. ATCC BAA-646 and *Haloferax* sp. BAB-2207, were available through NCBI (http://www.ncbi.nlm.nih.gov/genome/). All comparative genomic analyses were performed with the CGView Server^52^ using the default parameters (http://stothard.afns.ualberta.ca/cgview_server/).

## Additional information

**Accession codes:** The DNA microarray and high-throughput sequencing data have been deposited in the NCBI GEO library under accession numbers GSE70597 and GSE70592.

**How to cite this article:** Yang, H. *et al*. Activation of a dormant replication origin is essential for *Haloferax mediterranei* lacking the primary origins. *Nat. Commun.* 6:8321 doi: 10.1038/ncomms9321 (2015).

## Supplementary Material

Supplementary Information Supplementary Figures 1-7, Supplementary Tables 1-2 and Supplementary References

## Figures and Tables

**Table 1:** Strains used in this study.

***H. mediterranei*** **strains**	**Relevant characteristics**	**Source or reference**
DF50	Δ*pyrF* strain of *H. mediterranei* ATCC 33500	Liu *et al*.^26^
DFA50	*trpA* deletion mutant of DF50	This study
DF50Δ*oriC1-cdc6A*	*oriC1-cdc6A* deletion mutant of DF50	This study
DF50Δ*oriC2-cdc6C*	*oriC2-cdc6C* deletion mutant of DF50	This study
DF50Δ*oriC3-cdc6G*	*oriC3-cdc6G* deletion mutant of DF50	This study
DF50Δ*oriC4-cdc6H*	*oriC4-cdc6H* deletion mutant of DF50	This study
DF50Δ*oriP1-cdc6L*	*oriP1-cdc6L* deletion mutant of DF50	This study
DF50Δ*oriP2-cdc6M*	*oriP2-cdc6M* deletion mutant of DF50	This study
DF50Δ*oriC1*	*oriC1* deletion mutant of DF50	This study
DF50Δ*oriC2*	*oriC2* deletion mutant of DF50	This study
DF50Δ*oriC3*	*oriC3* deletion mutant of DF50	This study
DF50Δ*oriC4*	*oriC4* deletion mutant of DF50	This study
DF50Δ*oriC1*Δ*oriC2*	*oriC1* and *oriC2* deletion mutant of DF50	This study
DF50Δ*oriC1*Δ*oriC3*	*oriC1* and *oriC3* deletion mutant of DF50	This study
DF50Δ*oriC1*Δ*oriC2*Δ*oriC3*	*oriC1*, *oriC2* and *oriC3* deletion mutant of DF50	This study
DF50Δ*oriC1*Δ*oriC2*Δ*oriC4*	*oriC1*, *oriC2* and *oriC4* deletion mutant of DF50	This study
DF50Δ*oriC1*Δ*oriC3*Δ*oriC4*	*oriC1*, *oriC3* and *oriC4* deletion mutant of DF50	This study
DFA50Δ*oriC1*Δ*oriC2*Δ*oriC3*	*oriC1*, *oriC2, oriC3* and *trpA* deletion mutant of DF50	This study
DFA50Δ*oriC4::trpA*	Δ*oriC4::trpA* mutant of DFA50	This study
DF50R	p.tna-*radA* mutant of DF50	This study
DF50Δ*oriC1*Δ*oriC2*Δ*oriC3*R	p.tna-*radA* mutant of DF50Δ*oriC1*Δ*oriC2*Δ*oriC3*	This study
H13	Δ*oriC4-cdc6H::trpA-oriP2-orc13* mutant of DFA50Δ*oriC1*Δ*oriC2*Δ*oriC3*	This study

**Table 2:** Distribution of *H. mediterranei*-type replication origins in *Haloferax* species.

	**12** ***Haloferax*** species*	***Haloferax larsenii***	***Haloferax elongans***
*oriC1-cdc6A*	+	+	+
*oriC2-cdc6C*	+	+	+
*oriC3-cdc6G*	+	+	+
*oriC4-cdc6H*	—	+	+

^*^12 *Haloferax* species: *H. volcanii*, *H. sulfurifontis*, *H. mucosum*, *H. denitrificans*, *H. prahovense*, *H. gibbonsii*, *H. alexandrinus*, *H. lucentense*, *Haloferax* sp. ATCC BAA-644, *Haloferax* sp. ATCC BAA-645, *Haloferax* sp. ATCC BAA-646 and *Haloferax* sp. BAB-2207.

**Table 3:** Isolation of *oriC4* knockouts in different *H. mediterranei* strains.

**Starting strain**	**Knockouts of** ***oriC4*****/colonies analysed**
DF50	8/14
DF50Δ*oriC1*Δ*oriC3*	3/42
DF50Δ*oriC1*Δ*oriC2*Δ*oriC3*	0/(>600)
