# Supplementary material for: Activation of a dormant replication origin is essential for Haloferax mediterranei lacking the primary origins
Source: Nat Commun. 2015 Sep 16;6:8321. doi: 10.1038/ncomms9321 (PMC4595724; doi:10.1038/ncomms9321)
Supplement: Supplementary Information — Supplementary Figures 1-7, Supplementary Tables 1-2 and Supplementary References [file ncomms9321-s1.pdf]

Supplementary Figures

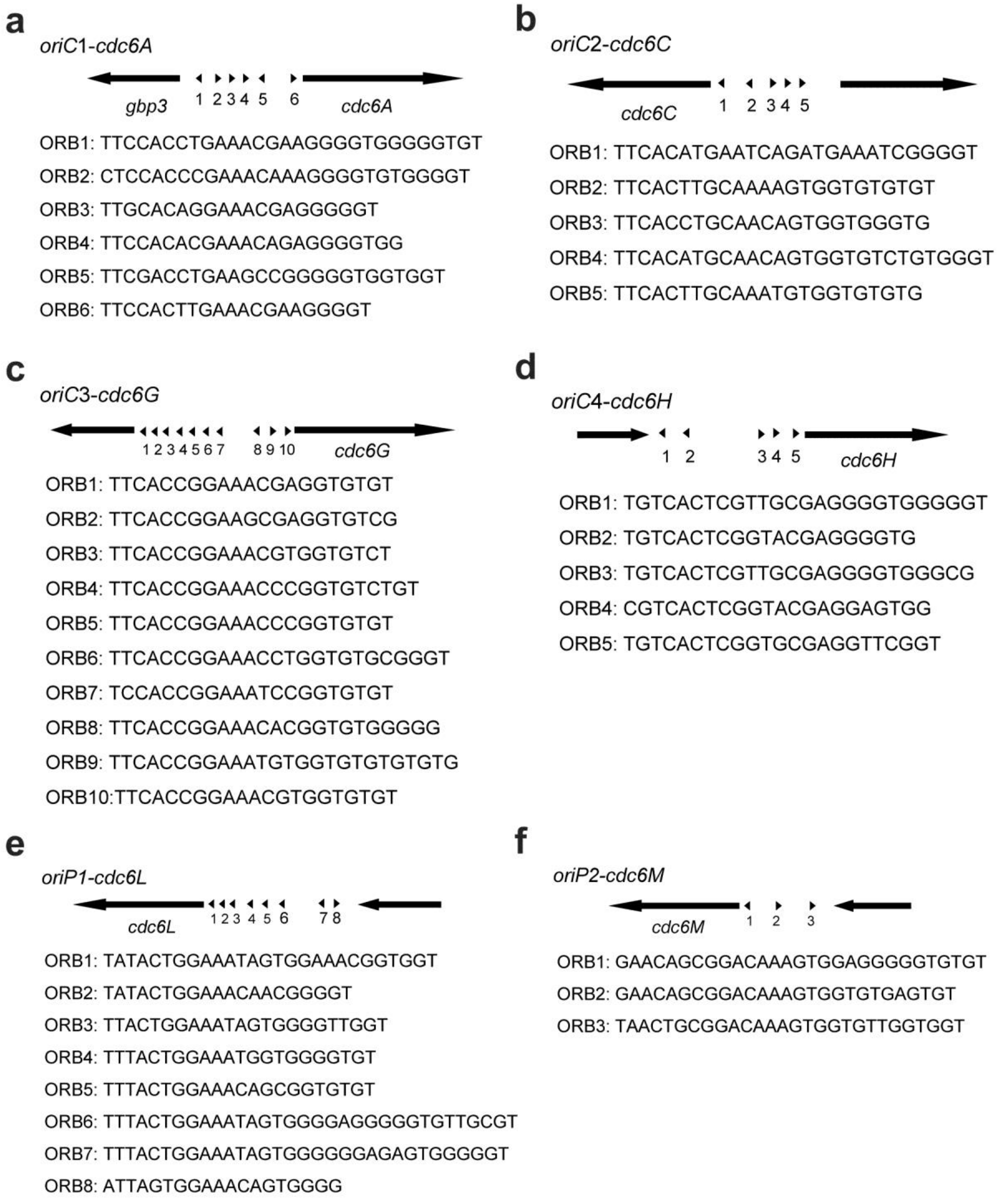

Supplementary Figure 1 | The ORB elements of the replication origins on the chromosome and on pHM500. The transcriptional direction of the genes flanking the origins is indicated by arrows, and the orientation of the ORBs within the origins is indicated by triangles.

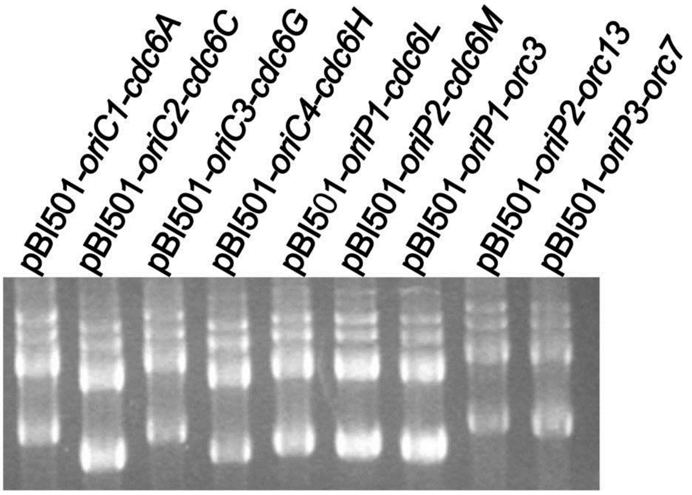

Supplementary Figure 2 | Retransferring the ARS plasmids from *H. mediterranei* transformants back to *E. coli*. The corresponding *H. mediterranei* transformant collected from the plate was lysed with 200 μL double-distilled H_2O and 100 μL phenol-chloroform, and the extracted plasmid in the supernatant was transferred into *E. coli*. The ARS plasmids were then extracted from *E. coli* and analysed by agarose gel electrophoresis.

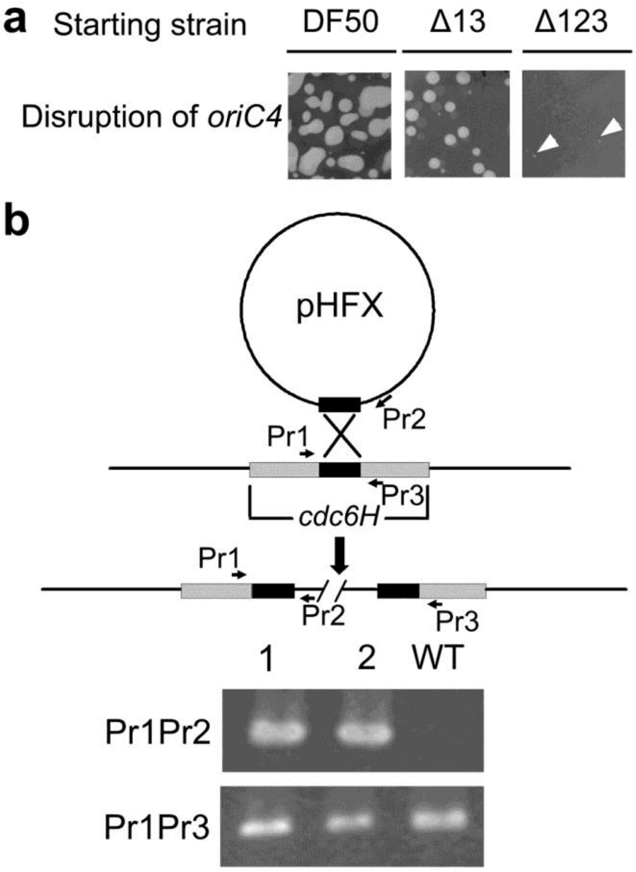

Supplementary Figure 3 | The disruption of *oriC4-cdc6H* in DF50, DF50Δ*oriC1*Δ*oriC3* and DF50Δ*oriC1*Δ*oriC2*Δ*oriC3*. (a) Transformation of pHFX-*cdc6H*-dis (Supplementary Table 1) into DF50, DF50Δ*oriC1*Δ*oriC3* (Δ13) and DF50Δ*oriC1*Δ*oriC2*Δ*oriC3* (Δ123). Colonies were observed after 4-5 days at 37°C. Disruption of *oriC4-cdc6H* in DF50Δ*oriC1*Δ*oriC2*Δ*oriC3* only resulted in sporadic and tiny colonies (indicated with white arrowheads), compared to those of DF50 and DF50Δ*oriC1*Δ*oriC3*. (b) PCR analysis of the disruption of *oriC4-cdc6H*. Upper panel: a schematic diagram of the disruption of *oriC4-cdc6H* via the single crossover method. Primers Pr1 and Pr2 were designed to confirm the integration of pHFX-*cdc6H*-dis at the *cdc6H* locus, and Pr1 and Pr3 were designed to determine the presence or absence of intact *cdc6H* genes (Supplementary Table 1). Lower panel: PCR analyses of chromosomal DNA prepared from the tiny colonies resulting from the disruption of *oriC4-cdc6H* in DF50Δ*oriC1*Δ*oriC2*Δ*oriC3*. Lanes 1, 2 represent transformants of DF50Δ*oriC1*Δ*oriC2*Δ*oriC3* and lane WT represents DF50. The PCR results are similar for the disruption of *oriC4-cdc6H* in DF50 and DF50Δ*oriC1*Δ*oriC3* (not shown).

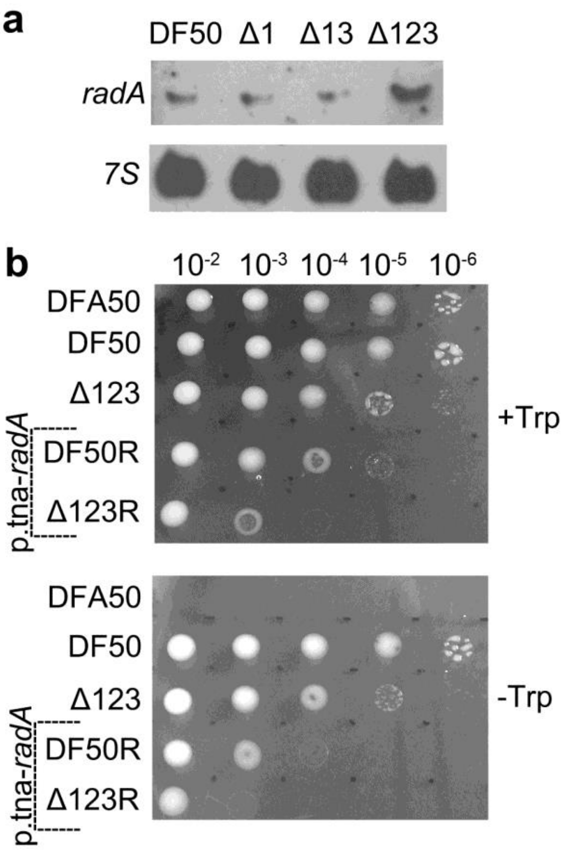

Supplementary Figure 4 | RadA is important but not essential for the growth of DF50Δ*oriC1*Δ*oriC2*Δ*oriC3*. (a) Northern blot analysis of the *radA* transcript in DF50, DF50Δ*oriC1* (Δ1), DF50Δ*oriC1*Δ*oriC3* (Δ13) and DF50Δ*oriC1*Δ*oriC2*Δ*oriC3* (Δ123). The *7S* transcripts are served as internal control. The total RNA was extracted from *H. mediterranei* during the exponential phase (OD_{600}≈0.35) with the TRIzol reagent. Northern blot analysis was performed as previously described[1]. The probes specific to *radA* and *7S* RNA were labeled with Biotin-11-dUTP (Thermo Scientific, R0081) by PCR. The signals were detected with the chemiluminescent nucleic acid detection module according to the manufacturer's protocol (Thermo Scientific, Rockford, USA). (b) Serial dilutions of stationary phase cells (in equal amounts) were spotted onto AS-168SY plates (supplemented with uracil) with (+Trp) or without (-Trp) tryptophan. *radA* was placed under the control of the tryptophan-inducible p.tna promoter in DF50R and DF50Δ*oriC1*Δ*oriC2*Δ*oriC3*R (Δ123R). DFA50 (auxotrophic for tryptophan), DF50 and DF50Δ*oriC1*Δ*oriC2*Δ*oriC3* (Δ123) were used as controls. The growth of DF50R and DF50Δ*oriC1*Δ*oriC2*Δ*oriC3*R were seriously affected by the absence of tryptophan but not to the lethal degree. The t.L11e terminator associated with p.tna promoter was placed upstream of *radA* ORF via double-crossover method with plasmid pBI501-ptnaradA (Supplementary Table 1).

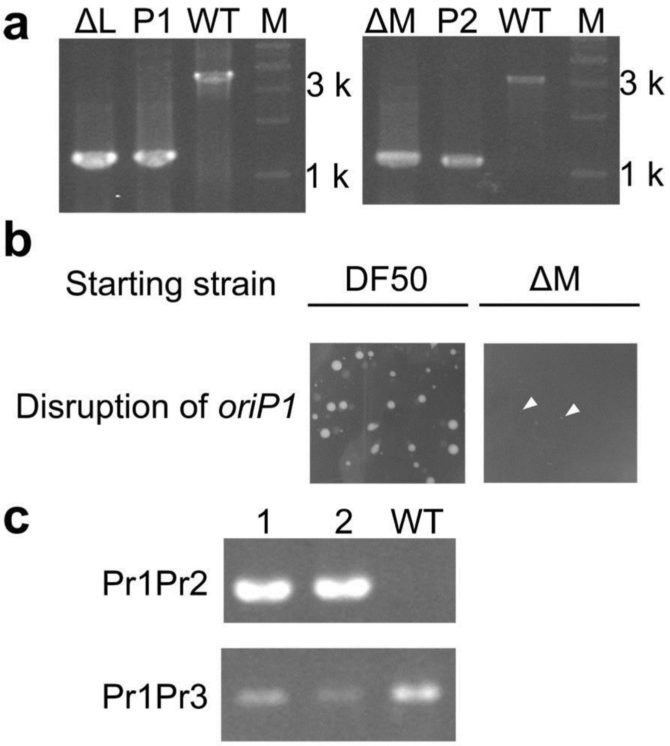

Supplementary Figure 5 | Importance of the *oriP1-cdc6L* for cell growth in the absence of *oriP2-cdc6M*. (a) Origin deletion strains were confirmed by PCR analyses. Lanes ΔL and ΔM represent the knockout of *oriP1-cdc6L* and *oriP2-cdc6M*, respectively; lanes P1 and P2 represent the plasmids used for deleting *oriP1-cdc6L* and *oriP2-cdc6M*, respectively; lane WT represents DF50. (b) Disruption of *oriP1-cdc6L* in DF50 and DF50Δ*oriP2-cdc6M*. Transformation of pHFX-*cdc6L*-dis (Supplementary Table 1) into DF50 and DF50Δ*oriP2-cdc6M* and colonies were observed after 4-5 days at 37°C. Disruption of *oriP1-cdc6L* in DF50Δ*oriP2-cdc6M* resulted in sporadic and tiny colonies (indicated with white arrowheads), compared to that of DF50. (c) PCR analyses of the chromosomal DNA prepared from the tiny colonies resulting from disruption of *oriP1-cdc6L* in DF50Δ*oriP2-cdc6M*. Lanes 1 and 2 represent transformants of DF50Δ*oriP2-cdc6M*, and lane WT represents DF50. The position of primers Pr1, Pr2, and Pr3 and the rationale for the primer design were similar to that of Supplementary Fig. 3b (Supplementary Table 1).

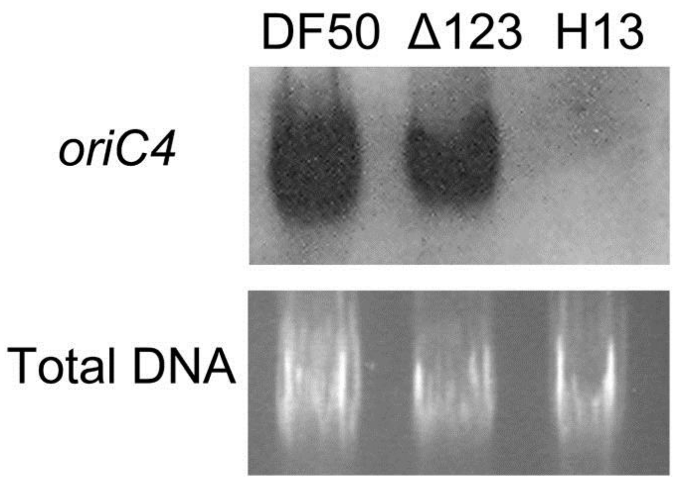

Supplementary Figure 6 | Confirmation of the absence of *oriC4* in H13 by Southern blot analysis with an *oriC4*-specific probe. DF50 and DF50Δ*oriC1*Δ*oriC2*Δ*oriC3* (Δ123) were selected as positive controls for *oriC4*. The total DNA was shown as loading control.

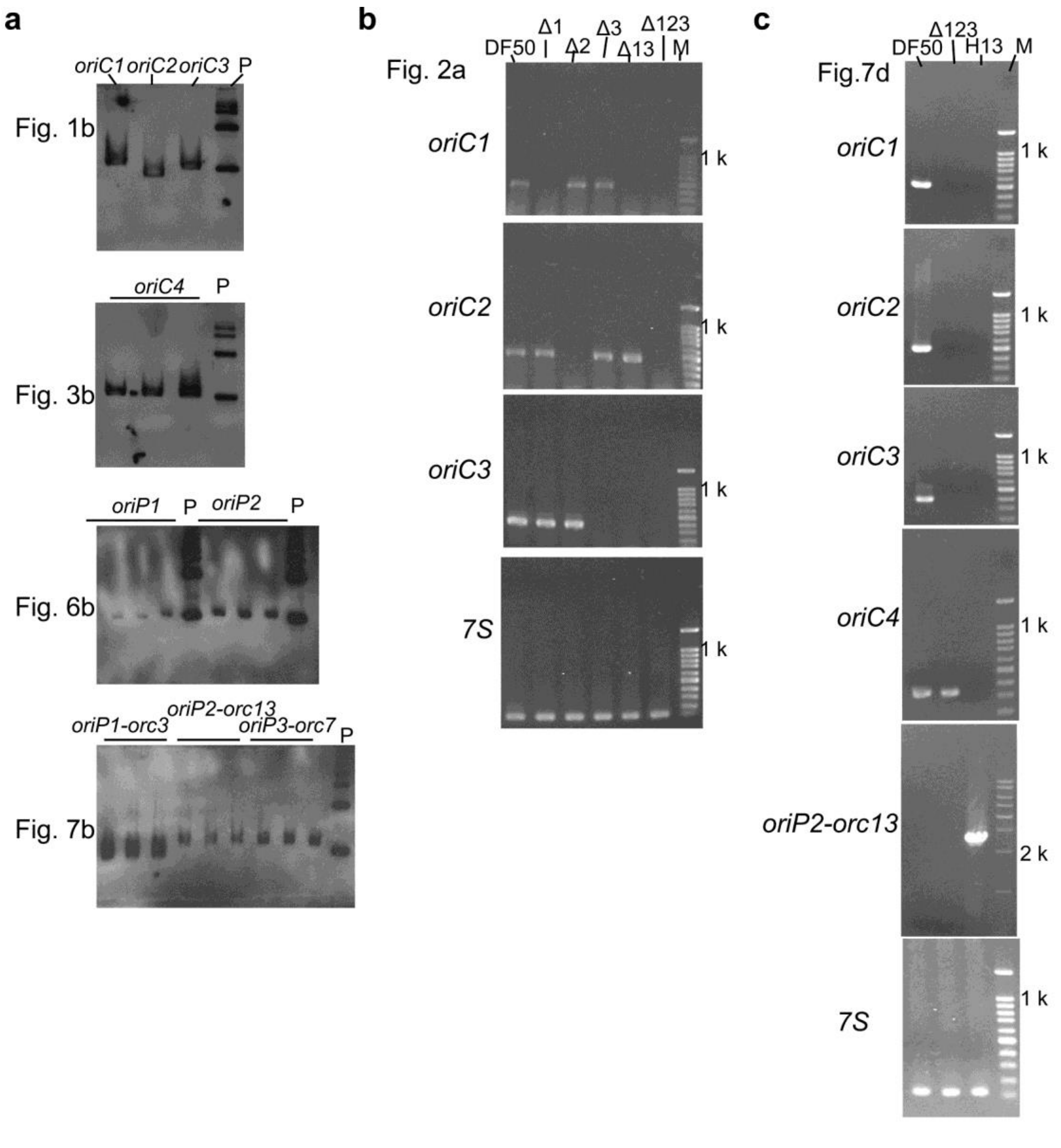

Supplementary Figure 7 | Full-length, uncropped Southern blot and agarose gel electrophoresis images for Figures 1 to 7. (a) Full length, uncropped images of Southern blot for figures 1b, 3b, 6b, and 7b. (b) Full length, uncropped images of agarose gel electrophoresis for Fig. 2a. (c) Full length, uncropped images of agarose gel electrophoresis for Fig. 7d.

Supplementary Tables

Supplementary Table 1. Oligonucleotides and constructed plasmids

Plasmids and oligonucleotides for knockout experiments			
Plasmid	Primer position	Primer name	Sequence (5'-3')*
pHFXΔ*oriC1-cdc6A*	5' forward	AFP1-PstI	AAAA**CTGCAG**ACCTTCGTCGTGACACCC
	5' reverse	AFP2-BamHI	CGC**GGATCC**CGAACAGCGTCAGGAGAA
	3' forward	APP3-BamHI	CGC**GGATCC**TGGAGTGTCGGAGTGGACT
	3' reverse	APP4-KpnI	ATAG**GGTACC**GAGAAGGGCTCAGAAGTCG
pHFXΔ*oriC2-cdc6C*	5' forward	CFP1-PstI	AAAA**CTGCAG**CTCCTCACGGGCATCTG
	5' reverse	CFP2-BamHI	CGC**GGATCC**TCAACAAAATAACCGAAACG
	3' forward	CPP1-BamHI	CGC**GGATCC**GCCTCGCTCAGAAACCC
	3' reverse	CPP2-KpnI	ATAG**GGTACC**AGCAGGAGACCGAACACC
pHFXΔ*oriC3-cdc6G*	5' forward	GFP1-PstI	AAAA**CTGCAG**AGAAAGTGCTGGGTTCGC
	5' reverse	GFP2b-BamHI	CGC**GGATCC**AGTTCTGCCGACTCATCTCTA
	3' forward	GPP3-BamHI	CGC**GGATCC**GACCGACCCAGTCACTACTTT
	3' reverse	GPP4-KpnI	ATAG**GGTACC**CAGAGCGAATGCGTGAAT
pHFXΔ*oriC4-cdc6H*	5' forward	HFP3-BamHI	CGC**GGATCC**GGTTCGGGTGAAGGACAG
	5' reverse	HFP4	CTACCACAACATCTCGAAGGATGGTTCGCTGAATA
	3' forward	HPP1’	AGCGAACCATCCTTCGAGATGTTGTGGTAGCTCCTG
	3' reverse	HPP2-KpnI	ATAG**GGTACC**GACCGCGATGCCTGATCT
pHFXΔ*oriP1-cdc6L*	5' forward	LFP1-PstI	AAAA**CTGCAG**AATCACCTCCTCGTTCGG
	5' reverse	LFP2	CGCTCTTGTATCTACTCCTACGGAACCCAACACT
	3' forward	LPP1	TTGGGTTCCGTAGGAGTAGATACAAGAGCGGCACG
	3' reverse	LPP2-BamHI	CGC**GGATCC**GTTGACACTGAGACCGCATC
pHFXΔ*oriP2-cdc6M*	5' forward	MFP1-PstI	AAAA**CTGCAG**GTTCTCGCCTTCAGTGTCG
	5' reverse	MFP2-BamHI	CGC**GGATCC**GGAAACCACGGAAGAGCC
	3' forward	MPP1-BamHI	CGC**GGATCC**CAACGAAATACCTTGTTGACC
	3' reverse	MPP2-KpnI	ATAG**GGTACC**CGATGGCAGTTGTAGCGT
pHFXΔ*oriC1*	5' forward	the same as AFP1-PstI	
	5' reverse	the same as AFP2-BamHI	
	3' forward	APP1-BamHI	CGC**GGATCC**TCATCTGGAATCGCCTTCT
	3' reverse	APP2-KpnI	ATAG**GGTACC**CTGCGAGGTAGACTCCAGTT
pHFXΔ*oriC2*	5' forward	CFP3-PstI	AAAA**CTGCAG**ACCGATGAGACCGACTTTC
	5' reverse	CFP4-BamHI	CGC**GGATCC**CACCCTGATGAATGGAACC
	3' forward	the same as CPP1-BamHI	
	3' reverse	the same as CPP2-KpnI	
pHFXΔ*oriC3*	5' forward	the same as GFP1-PstI	
	5' reverse	the same as GFP2b-BamHI	
	3' forward	GPP1a-BamHI	CGC**GGATCC**TCTCCACTTCTCCCATCTTG
	3' reverse	GPP2-KpnI	ATAG**GGTACC**TGACGCCGAGTTTACAGC

pHFXΔ*oriC4*	5' forward	HFP1-PstI	AAAA**CTGCAG**CGAGGATGACCACGAAGAC
	5' reverse	HFP2-BamHI	CGC**GGATCC**GGTCGTCAACATAGAATCACA
	3' forward	HPP1-BamHI	CGC**GGATCC**GAGATGTTGTGGTAGCTCCTG
	3' reverse	the same as HPP2-KpnI	
pHFXΔ*trpA*	5' forward	trpAFP1-BamHI	CGC**GGATCC**GCAAGCCCACGAGCAGAT
	5' reverse	trpAFP2	AGGGTGACAGAGCAGATTACAGTCCCCCCGTGAAC
	3' forward	trpAPP1	CGGGGGGACTGTAATCTGCTCTGTCACCCTTTCTC
	3' reverse	trpAPP2-KpnI	ATAG**GGTACC**CGTCGTGCTGGAAGATAGAC
Plasmids and oligonucleotides for *trpA*-marked positive selection system			
Plasmid	Description	Primer name	Sequence (5'-3')*
pBI501	pUCmT[2] with insertion of *pyrF* marker at EcoR I-Kpn I sites		
pSCM307::*trpA*	pSCM307[3] with insertion of *trpA* at the 3' end of the *hsp5* promoter	trpAFP-NdeI	GGAATTC**CATATG**ATGTCGCTCGAAGACGCC
		trpARP-PstI	AAAA**CTGCAG**TTATGTGCGTTCCGAATGC
pHFA101	pBI501 with insertion of *Phsp5-trpA* at BamHI-NcoI sites	hsptrpAFP-BamHI	AAT**GGATCC**TCCGTCTGGGAATTCGAT
		hsptrpARP-NcoI	CATG**CCATGG**TTATGTGCGTTCCGAATGC
pHFA101Δ*oriC4*	pHFA101 with insertion of upstream and downstream fragments of *oriC4* flanking *Phsp5-trpA*	the same as HFP1-PstI	
		the same as HFP2-BamHI	
		HPP1-NcoI	CATG**CCATGG**GAGATGTTGTGGTAGCTCCTG
		the same as HPP2-KpnI	
pHFA101-Δ*oriC4-cdc6H::oriP2-orc13*	amplification of upstream fragment of *oriC4-cdc6H*	HFP1a-XbaI	CTAG**TCTAGA**GAAGGAGGATACTGGTTGGG
		HFP2-BamHI	CGC**GGATCC**GGTCGTCAACATAGAATCACA
	amplification of *oriP2-orc13*	orc13+FP-NcoI	CATG**CCATGG**GGACGGCACATCACATACAA
		orc13+FP2-EcoRV	CTGC**GATATC**GCTGGGATCGGAATGATG
	amplification of downstream fragment of *oriC4-cdc6H*	HPP1-EcoRV	CTGC**GATATC**GAGATGTTGTGGTAGCTCCTG
		HPP2a-KpnI	ATAG**GGTACC**CTCCAACTGTTCGCCTACC
Plasmids and oligonucleotides for *radA* promoter replacement			
Plasmid	Description	Primer name	Sequence (5'-3')*
pTA1228-*radA*	pTA1228[4] with	radA-FP-NdeI	GGAATTC**CATATG**GCAGAAGACGACCTCGAAAAT

	radA at the downstream of p.tna promoter	radA-RP-NotI	ATAAGAAT**GCGGCCGC**AATCTATTCGGGCTTGAGACC
pBI501-ptnaradA	amplification of upstream fragment of *radA* promoter	radAFP1-PstI	AAAA**CTGCAG**CGCCTTCTCGCTGCTCTC
		radAFP2-BamHI	CGC**GGATCC**CCGCCCATACCCACATAA
	amplification of t.L11e &p.tna-*radA* from pTA1228-*radA*	ptna-radAFP-BamHI	CGC**GGATCC**ACTCACTATAGGGCGAATTGG
		ptna-radARP-NcoI	CATG**CCATGG**AGGAGAATCTGGTGGTTGGA

Plasmids and oligonucleotides for gene disruption experiments		
Plasmid	Primer name	Sequence (5'-3')*
pHFX-*cdc6H*-dis	HFPdis-BamHI	CGC**GGATCC**AGGTCTTGGAAACGCTTGA
	HRPdis-KpnI	ATAG**GGTACC**CGACGAACTCGCTCCATC
pHFX-*cdc6L*-dis	LFP2dis-BamHI	CGC**GGATCC**TCGTGTTCGGTGAGGTCG
	LRP2dis-KpnI	ATAG**GGTACC**TCCGCAGTCCAAGGTCTAT

Plasmids and oligonucleotides for ARS assays		
Plasmid	Primer name	Sequence (5'-3')*
pBI501-*oriC1-cdc6A*	Cdc6A+FP1-BamHI	CGC**GGATCC**TGTTTCGTCTCACCTTTCCT
	Cdc6A+PP2-KpnI	ATAG**GGTACC**GTCCGGTAATCCGTATCTCG
pBI501-*oriC2-cdc6C*	Cdc6C+FP2-BamHI	CGC**GGATCC**TTGACTCAGACCGCTTCGG
	OriC2FP2-KpnI	ATAG**GGTACC**TCGAGGCTATTCAAAGGGA
pBI501-*oriC3-cdc6G*	Cdc6G+FP1-BamHI	CGC**GGATCC**CCTGCTTGTGGTGCTTCTC
	Cdc6G+PP1-NcoI	CATG**CCATGG**GTCACAAAATACCGCAACAA
pBI501-*oriC4-cdc6H*	Cdc6H+FP1-BamHI	CGC**GGATCC**CGAGGGTCTGGATTAGTTTC
	Cdc6H+PP1-KpnI	ATAG**GGTACC**TCGTCGAATCTCGAACCG
pBI501-*oriC4*	oriC4-FP-BamHI	CGC**GGATCC**GCTACCGAACCTCGCACC
	Cdc6H+PP1-KpnI	ATAG**GGTACC**TCGTCGAATCTCGAACCG
pBI501-*cdc6H*	Cdc6H+FP1-BamHI	CGC**GGATCC**CGAGGGTCTGGATTAGTTTC
	Cdc6H-RP-KpnI	ATAG**GGTACC**GGTCGTCAACATAGAATCACA
pBI501-*oriP1-cdc6L*	Cdc6L+FP-BamHI	CGC**GGATCC**GTCATTTCAGGTCTGGTATCG
	Cdc6L+PP-NcoI	CATG**CCATGG**CCTCGTATCCATCCTGCTC
pBI501-*oriP2-cdc6M*	Cdc6M+FP-BamHI	CGC**GGATCC**CGGTGGGTATCCTCATCC
	Cdc6M+PP-NcoI	CATG**CCATGG**GAACTCTGCTGTGGACGGA
pBI501-*oriP1-orc3*	orc3+FP-BamHI	CGC**GGATCC**AGACGAGTCGGCTGGTGA
	orc3+RP-NcoI	CATG**CCATGG**TTCCGCAGAGCAGGTCAA
pBI501-*oriP2-orc13*	orc13+FP-NcoI	CATG**CCATGG**GGACGGCACATCACATACAA
	orc13+RP-KpnI	ATAG**GGTACC**GCTGGGATCGGAATGATG
pBI501-*oriP3-orc7*	orc7+FP-NcoI	CATG**CCATGG**GGTGTGGGTTACCAGACTGAA
	orc7+RP-KpnI	ATAG**GGTACC**GCAGGGCTTGGAACAGG
pBI501-*orc4+*	orc4+FP-NcoI	CATG**CCATGG**GGGAGGAAGGAAGTCGG
	orc4+RP-KpnI	ATAG**GGTACC**CTGTTCTGTGGGTTACTGCTC
Oligonucleotides for detection of gene disruption		

Primer name	Sequence (5'-3')*
cdc6Hdis-Pr1	the same as Cdc6H+FP1-BamHI
cdc6Hdis-Pr2	ATCGCCTTCGGGAAGACT
cdc6Hdis-Pr3	GCGAAGACCAGGGAGAAC
cdc6Ldis-Pr1	the same as Cdc6L+FP-BamHI
cdc6Ldis-Pr2	ATAG**GGTACC**CGAAGCAGCCCTACAACC
cdc6Ldis-Pr3	the same as cdc6Hdis-Pr3
Oligonucleotides for detection of origins	
Primer name	Sequence (5'-3')*
oriC1-F	CGC**GGATCC**TGTTTCGTCTCACCTTTCCT
oriC1-R	CATG**CCATGG**GAAATCGGCGGGTAGAAT
oriC2-F	CGC**GGATCC**GGTTCCATTCATCAGGGTG
oriC2-R	ATAG**GGTACC**TCGAGGCTATTCAAAGGGA
oriC3-F	CGC**GGATCC**CCTGCTTGTGGTGCTTCTC
oriC3-R	TCCTTTGAACCCGACTTCTC
oriC4-F	TGGATCGAGAGTCGACAGCT
oriC4-R	CAGTCTCGCTTGGAGTGTTG
oriP2-orc13-F	the same as orc13+FP-NcoI
oriP2-orc13-R	the same as orc13+FP2-EcoRV
7S-F	ACTAGGTCGGGCAGTTAGG
7S-R	CGAAGGACGAGGTTTCTACG

* Sequences representing restriction sites are highlighted in bold;

Supplementary Table 2. Location of putative origins from *H. volcanii* surveyed in our study

Putative origins	Replicon in wild isolate DS2	Replicon in laboratory strain H26	Location[5]
oriP1-orc3	pHV4	chromosome	635565-635786&1-1533*
oriP2-orc13	pHV4	chromosome	55221-57786*
oriP3-orc7	pHV4	chromosome	257317-259691*
orc4+	chromosome	chromosome	1887008-1889595*

* Numbering refers to the pHV4 or chromosome position of *H. volcanii* DS2.

Supplementary References

1. Liu X, Wang L, Liu J, Cai L, Xiang H. Genome-wide analysis of gene expression in stationary phase and genetic characterization of stationary-phase-dependent halocin gene expression in the haloarchaeon *Haloferax mediterranei*. *J. Genet. Genomics* **40,** 441-444 (2013).

2. Sun CM, Zhou MX, Li Y, Xiang H. Molecular characterization of the minimal replicon and the unidirectional theta replication of pSCM201 in extremely halophilic archaea. *J. Bacteriol.* **188,** 8136-8144 (2006).

3. Miao D, Sun C, Xiang H. [Construction and application of a novel shuttle expression vector based on haloarchaeal plasmid pSCM201]. *Wei Sheng Wu Xue Bao* **49,** 1040-1047 (2009).

4. Brendel J, *et al.* A complex of Cas proteins 5, 6, and 7 is required for the biogenesis and stability of clustered regularly interspaced short palindromic repeats (CRISPR)-derived RNAs (crRNAs) in *Haloferax volcanii*. *J. Biol. Chem.* **289,** 7164-7177 (2014).

5. Hartman AL, *et al.* The complete genome sequence of *Haloferax volcanii* DS2, a model archaeon. *PLoS One* **5,** e9605 (2010).
